# Preparation of Fibrinogen-Depleted Human Platelet Lysate to Support Heparin-Free Expansion of Umbilical Cord-Derived Mesenchymal Stem Cells

**DOI:** 10.3390/biology12081085

**Published:** 2023-08-03

**Authors:** Li Ting Kee, Yi Ting Lee, Chiew Yong Ng, Muhammad Najib Fathi Hassan, Min Hwei Ng, Zalina Mahmood, Suria Abdul Aziz, Jia Xian Law

**Affiliations:** 1 Centre for Tissue Engineering and Regenerative Medicine, Faculty of Medicine, Universiti Kebangsaan Malaysia, Cheras, Kuala Lumpur 56000, Malaysia; liting1027@hotmail.com (L.T.K.); chiewyongng@gmail.com (C.Y.N.); najibfathi93@gmail.com (M.N.F.H.); angela@ppukm.ukm.edu.my (M.H.N.); 2 Department of Physiology, Faculty of Medicine, Universiti Kebangsaan Malaysia, Cheras, Kuala Lumpur 56000, Malaysia; yitingyyl428@gmail.com; 3 National Blood Centre of Malaysia, Kuala Lumpur 50400, Malaysia; dr.zalina@moh.gov.my; 4 Department of Pathology, Faculty of Medicine, Universiti Kebangsaan Malaysia, Cheras, Kuala Lumpur 56000, Malaysia; suria.abdulaziz@ppukm.ukm.edu.my

**Keywords:** human platelet lysate, calcium, fibrinogen, mesenchymal stem cell, cell culture

## Abstract

**Simple Summary:**

The use of fetal bovine serum (FBS) in the manufacturing and production of therapeutic products is discouraged by the regulatory authorities due to the risk of zoonoses and xenogeneic immune reactions. Human platelet lysate (hPL) is suggested as an alternative to FBS for in vitro cell expansion in accordance with good manufacturing practice (GMP) regulations. Since hPL contains a lot of fibrinogen and other coagulation factors, heparin is commonly added to prevent gel formation. However, heparin is typically derived from animals, and despite following the recommended maximum dose, gel and precipitate formation can still occur during storage and cell culture. Thus, an alternative preparation method is proposed in this study to prepare fibrinogen-depleted hPL (Fd-hPL) that supports heparin-free expansion of umbilical cord-derived mesenchymal stem cells (UC-MSCs). The findings revealed that Fd-hPL supported the in vitro expansion of UC-MSCs without compromising their growth and characteristics. Therefore, it has the potential to be used as a substitute for FBS for in the vitro culture of MSCs without introducing xenogeneic components. However, further research is necessary to fully understand the functionality and therapeutic potential of these expanded cells.

**Abstract:**

Human platelet lysate (hPL) has high levels of fibrinogen and coagulation factors, which can lead to gel and precipitate formation during storage and cell culture. Heparin derived from animals is commonly added to minimize these risks, but cannot completely eliminate them. Thus, this study proposes an alternative method to prepare fibrinogen-depleted hPL (Fd-hPL) that supports heparin-free expansion of mesenchymal stem cells (MSCs). hPL was added to heparin to prepare heparin-hPL (H-hPL), whilst Fd-hPL was prepared by adding calcium salt to hPL to remove the fibrin clot. The concentrations of calcium, fibrinogen, and growth factors in H-hPL and Fd-hPL were compared. The effects of H-hPL and Fd-hPL on umbilical cord-derived MSCs (UC-MSCs) were assessed. The results showed that Fd-hPL possessed a significantly higher calcium concentration and a lower fibrinogen level than H-hPL. The concentrations of BDNF, TGF-β1, and PDGF-BB showed no significant difference between H-hPL and Fd-hPL, but Fd-hPL had a lower VEGF concentration. Fd-hPL retained the characteristics of UC-MSCs, as it did not affect the cell viability, proliferation, multilineage differentiation potential, or surface marker expression. In conclusion, Fd-hPL effectively supported the in vitro expansion of MSCs without compromising their characteristics, positioning it as a potential substitute for FBS in MSC culture.

## 1. Introduction

Fetal bovine serum (FBS) is commonly used as a serum supplement for in vitro cell expansion as it provides growth factors, hormones, proteins, lipids, carbohydrates, electrolytes, and other undefined components, which are essential in supporting cell survival, attachment, metabolism, proliferation, and growth [1]. Furthermore, FBS maintains the stemness, specific surface markers, and multilineage differentiation potential of mesenchymal stem cells (MSCs) [2]. Due to its robustness, FBS is also widely used to culture many other cell types, including the primary cells and cell lines of human and animal origins. Nonetheless, FBS is not ideal for use in expanding cells meant for clinical use due to the risk of animal pathogen transmission and activation of the xenogeneic immune response in the transplanted host [3]. These disadvantages suggest that the use of FBS is not compliant with the good manufacturing practice (GMP) principle as it may affect the safety and efficacy of cell therapy [4]. Therefore, chemically defined serum-free medium and human serum have been explored as potential xenogeneic-free alternatives for FBS. The transition from ill-defined serum to a chemically defined medium may offer advantages. However, this switch can pose challenges, as different cell types may necessitate varying formulation adjustments. Consequently, the process becomes costly and may not be applicable or utilized in many situations [5].

Platelet concentrate is a type of blood component stored in blood banks for clinical use. In Malaysia, platelet concentrate is kept for five days at 20–24 °C under constant agitation in accordance with the Ministry of Health’s guidelines and is discarded as biological waste if no transfusion is made [6]. Constant agitation is critical to prevent clot formation, but this affects the platelet viability and functionality by causing platelet activation, resulting in a short shelf life [7]. Outdated platelet concentrates can be salvaged by processing into hPL for cell culture purposes as it still contains a high level of growth factors that are needed to sustain the viability and growth of cultured cells [8]. The cells cultured with hPL are safe for clinical applications as platelet activation does not produce any harmful by-products. In fact, outdated platelet concentrates are subjected to additional processing steps to achieve a higher rate of platelet activation to enhance the release of proteins and growth factors into the plasma. The use of expired concentrates to produce hPL has many advantages, such as no active blood taking from donors (to avoid competition with a blood bank who has a high demand for blood to save life) and no ethical issues [9]. Furthermore, platelet concentrates are collected from low-risk populations and screened for transfusion-transmissible infections to minimize the risk of transfusion recipient allosensitization, as well as transfusion-transmissible infections [10]. Hence, the cells cultured with hPL are considered safer compared to those expanded with FBS.

Many methods can be applied to lyse and release the growth factors from platelets to obtain cell-free and growth factor-rich hPL, such as repeated freezing and thawing [10], direct platelet activation using calcium salt or thrombin [11], sonication [12], solvent/detergent treatment [1], and the combination of these techniques [13]. Generally, heparin must be added to hPL-supplemented culture medium to prevent spontaneous gel formation due to the high fibrinogen concentration present in hPL. Heparin is a glycosaminoglycan with anti-coagulant activity to prevent the formation of fibrin clots [14]. Heparin has been reported to promote the expansion of human stem cells, whereby it increases the proliferation of human embryonic stem cells [15] and MSCs [16]. However, a high concentration of heparin is linked with suppressed MSC proliferation, altered cell morphology, and phenotype, as well as MSC senescent [14].

In our previous study, in-house hPL prepared from expired platelet concentrates was found to be more efficient than FBS in promoting the proliferation of human skin fibroblasts [17], chondrocytes [18,19], and umbilical cord-derived MSCs (UC-MSCs) [20]. However, clot formation was observed during storage and occasionally in medium preparation and culture flasks, even though a final concentration of 4 IU/mL of heparin was added to the complete medium. As a high concentration of heparin adversely affects cell functionality, the depletion of fibrinogen and other clotting factors is more ideal than further increasing the heparin concentration to abrogate clot formation.

In this study, calcium chloride was added into hPL prepared using the freeze/thaw cycle method to stimulate clot formation. Calcium ions mediated the conversion of prothrombin into thrombin and expedite the rate of fibrin monomer polymerization, thereby enhancing fibrin clot formation [21]. Then, the precipitate formed was removed by centrifugation to obtain fibrinogen-depleted hPL (Fd-hPL). The concentrations of calcium, fibrinogen, and growth factors were measured to evaluate the efficacy of the procedure, while the functionality of Fd-hPL was evaluated through the cultivation of UC-MSCs.

## 2. Materials and Methods

### 2.1. Human Platelet Lysate Preparation

**Figure 1:**
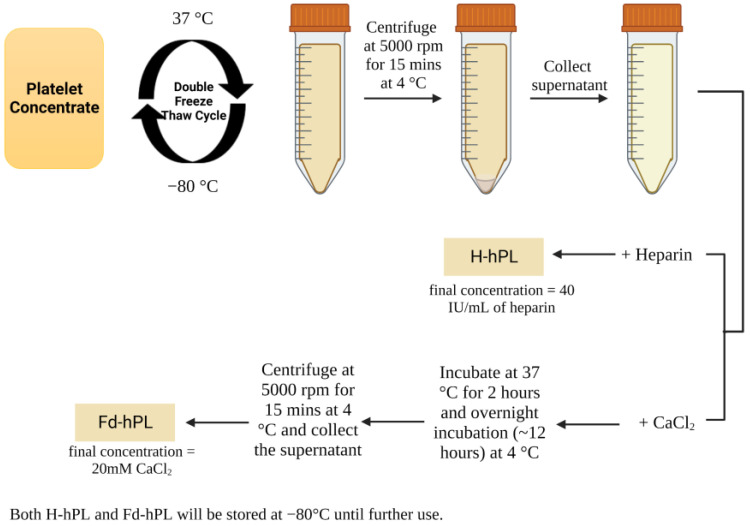
Preparation of hPL. Platelet concentrates were pooled and underwent repeated freeze–thaw cycling to prepare H-hPL through the addition of 40 IU/mL of heparin. Calcium chloride was added to hPL to stimulate clot formation, which was later removed via centrifugation to prepare Fd-hPL. Created with Biorender.com (accessed date: 20 July 2023).

Expired human platelet concentrates were collected from the Blood Bank Unit, Hospital Canselor Tuanku Muhriz, Kuala Lumpur, Malaysia (ethical approval: UKM PPI/111/8/JEP-2023-033). Briefly, the expired platelet concentrates were pooled and exposed to freeze–thaw cycles twice, at 37 and −80 °C (Figure 1). Then, the platelet concentrates were centrifuged at 5000 rpm for 15 min at 4 °C to separate the supernatant (hPL) that was collected and precipitate that which was discarded. H-hPL was prepared by adding 40 IU/mL of heparin (Duopharma, Malaysia) into the hPL. To prepare Fd-hPL, calcium chloride (CaCl_2_) (Sigma-Aldrich, St. Louis, MO, USA) was added into hPL to a final concentration of 20 mM and incubated at 37 °C for 2 h, followed by overnight incubation at 4 °C. After this, the clotted hPL was centrifuged at 5000 rpm for 15 min at 4 °C. The supernatant (Fd-hPL) was collected. Both H-hPL and Fd-hPL were stored at −80 °C until further use.

### 2.2. Calcium Assay

The calcium ion concentration in H-hPL and Fd-hPL was identified using a calcium colorimetric assay kit (Sigma-Aldrich, St. Louis, MO, USA) following the manufacturer’s protocol. Briefly, H-hPL and Fd-hPL were diluted with MilliQ water to 10× and 100×, respectively, and added into a 96-well-flat bottom plate. The chromogenic reagent was added into standard and sample wells, followed by adding the calcium assay buffer. The mixture was mixed gently and incubated in the dark at room temperature for 5–10 min. The absorbance was read at 575 nm using a spectrophotometric multi-well plate reader (Biotek^®^, Santa Clara, CA, USA) with Gen 5 software, version 2.09. 

### 2.3. Measurement of Fibrinogen and Growth Factor Concentration

An enzyme-linked immunosorbent assay (ELISA) was performed to measure the concentration of fibrinogen (Cusabio, Wuhan, China), BDNF (BioLegend, San Diego, CA, USA), VEGF (BioLegend, San Diego, CA, USA), PDGF-BB (Qayee-Bio, Shanghai, China), and TGF-β (Qayee-Bio, Shanghai, China). The assay was performed following the manufacturer’s protocol. Briefly, H-hPL and Fd-hPL were diluted with supplied diluent before adding to the antibody-coated wells and incubated for 1 to 2 h. Then, the detection antibody and HRP-avidin were added sequentially and incubated for 30 min to 1 h. After this, the TMB substrate solution was added to each well and incubated for 10–15 min at 37 °C in the dark, followed by the addition of stop solution. The absorbance was read at 450 nm within 5 min using a spectrophotometric multi-well plate reader.

### 2.4. Isolation and Culture of Umbilical Cord-Derived Mesenchymal Stem Cells (UC-MSCs)

The umbilical cord sample was collected from mothers undergoing caesarean section in Hospital Canselor Tuanku Muhriz with consent (ethical approval: UKM PPI/111/8/JEP-2023-033). Briefly, arteries and veins were removed from the umbilical cord before mincing into small pieces for enzymatic digestion using 0.6% collagenase type I (Worthington, Lakewood, NJ, USA) for 1 h at 37 °C in a shaking incubator. The isolated cells were cultured in low-glucose Dulbecco’s modified Eagle medium (LG-DMEM; Sigma-Aldrich, St. Louis, MO, USA) with 1% GlutaMAX™ (Gibco, Grand Island, NY, USA), 1% antibiotic–antimycotic (Capricorn, Ebsdorfergrund, Germany), and 10% FBS (Capricorn, Ebsdorfergrund, Germany), 10% H-hPL, or 10% Fd-hPL.

### 2.5. Mesenchymal Stem Cell Characterization

The UC-MSCs cultured with Fd-hPL were characterized according to the International Society for Cell and Gene Therapy (ISCT) guidelines [22]. The MSC positive and negative markers were identified using a Human MSC Analysis Kit (BD Biosciences, San Jose, CA, USA) following the manufacturer’s protocol. Briefly, 1 × 10^6^ cells were resuspended in 100 µL of phosphate saline buffer (PBS) + 1% FBS in microcentrifuge tubes. The cells were labeled with positive antibodies (CD90, CD105, and CD73) and negative antibodies (CD34, CD11b, CD19, CD4, and HLA-DR) in the dark at room temperature for 30 min. Then, the cells were washed with PBS + 1% FBS and analyzed on a flow cytometer (BD FACSVerse™, San Jose, CA, USA).

UC-MSCs were cultured in 12-well plates with StemPro™ adipogenesis and osteogenesis differentiation media (ThermoFisher Scientific, Grand Island, NY, USA) for 14 days. The differentiation media was changed every two to three days. After induction, the cells were fixed with 4% paraformaldehyde and stained with oil red O (Sigma-Aldrich, St. Louis, MO, USA) and Alizarin red (Sigma-Aldrich, St. Louis, MO, USA) to detect lipid droplets in adipogenic differentiated cells and calcium deposition in osteogenic differentiated cells, respectively.

### 2.6. Cell Morphology, Cell Size, Cell Viability, Total Cell Number, and Population Doubling Time Assessment

UC-MSCs at passage 4 were seeded in six-well plates with a seeding density of 3000 cells/cm^2^ in LG-DMEM with 1% GlutaMAX™ (Gibco, Grand Island, NY, USA), 1% antibiotic–antimycotic (Capricorn, Ebsdorfergrund, Germany), and 10% FBS (Capricorn, Ebsdorfergrund, Germany), H-hPL, or Fd-hPL. The morphological changes, growth pattern, and confluency of cultured UC-MSCs were observed and captured using an inverted microscope at a magnification of 40×. Five points per well were captured. The length, width, and size of the cells were analyzed using ImageJ software, version 1.53k. Then, the cells were trypsinized with 0.05% trypsin-EDTA (Gibco, Grand Island, NY, USA) when the cell confluency reached 70%–80%. The cells were stained with 0.4% trypan blue solution (Corning^®^, Manassas, VA, USA) and the number of viable and non-viable cells was counted using a hemocytometer. The population doubling time (PDT) was calculated using the following formula:

$$\mathrm{PDT}\;(\mathrm{hour})=\frac{t\log2}{\log N2-\log N1}$$

where t denotes time in culture; N2 denotes the cell number at the end of the passage; N1 denotes the cell number seeded at the beginning of the passage.

### 2.7. Statistical Analysis

Statistical analysis was conducted using GraphPad Prism version 9.4.1. The data are presented as the mean ± standard error of the mean (SEM). An unpaired *t*-test was used to analyze the significance between two groups, while the significance among three or more groups was examined using a one-way ANOVA. A *p*-value < 0.05 was considered statistically significant.

## 3. Results

### 3.1. Fd-hPL Has a Higher Calcium Concentration and a More Noticeable Trend with a Lower Fibrinogen Concentration Compared to H-hPL

**Figure 2:**
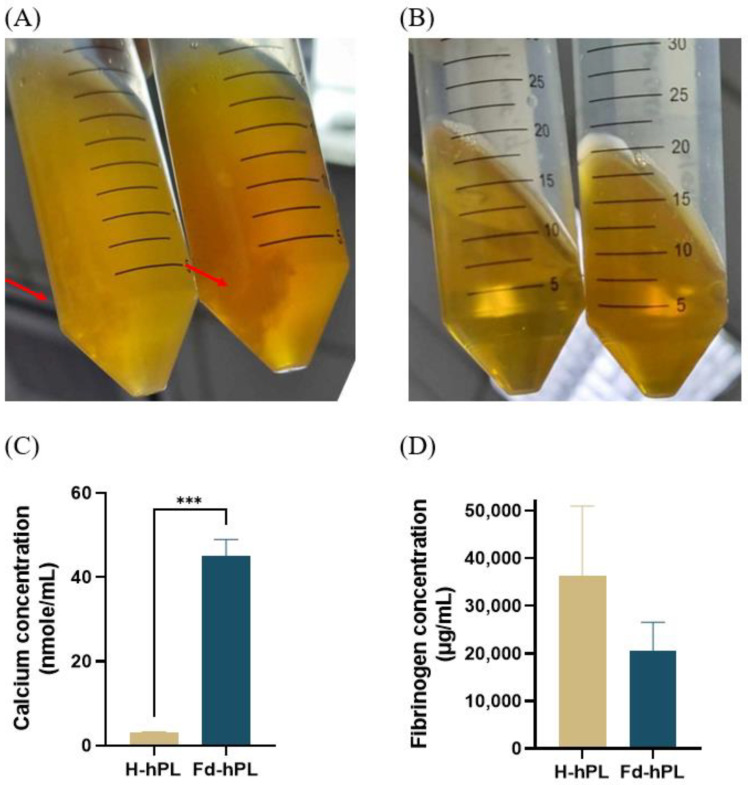
Picture of (**A**) H-hPL and (**B**) Fd-hPL after two times freeze-thaw cycles. Precipitate formation was observed in H-hPL and absent in Fd-hPL. Red arrow indicating the precipitates formed in hPL. (**C**) The calcium level in Fd-hPL was significantly higher than in H-hPL. (**D**) Fd-hPL showed a more noticeable trend with lower levels of fibrinogen compared to H-hPL. Values expressed as the mean ± SEM (*N* = 6 per group). *** *p* < 0.001; unpaired *t*-test with the Holm–Šídák method.

The existence of precipitate formation in the H-hPL was seen as Figure 2A, while Figure 2B reveals no visible precipitate in the Fd-hPL sample, showing that such precipitate forms did not exist. The calcium level in the Fd-hPL group (45.10 ± 3.89 nmole/mL) treated with 20 mM of CaCl_2_ was significantly higher compared to the H-hPL group (3.03 ± 0.24 nmole/mL) (*** *p* < 0.001) (Figure 2C). After the fibrinogen depletion process, the fibrinogen concentration of hPL reduced from 36,253 ± 18,996 to 20,644 ± 5955 μg/mL (Figure 2D). As a comparison, the fibrinogen concentration of two commercial hPL and the FBS was also measured. It was found that the fibrinogen levels of commercial hPL 1 and hPL 2 were 74,409 and 23,248 μg/mL, respectively. The fibrinogen level of FBS was below the detection limit of the kit.

### 3.2. The Concentration of VEGF Was Reduced Significantly in Fd-hPL Compared to H-hPL

**Figure 3:**
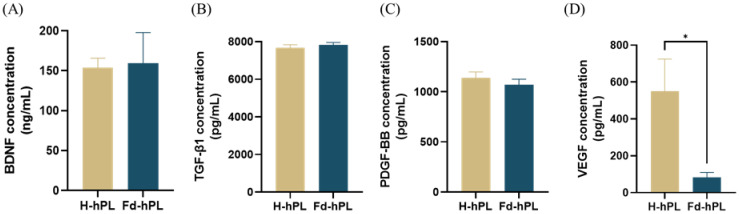
Concentrations of growth factors in H-hPL and Fd-hPL. (**A**) BDNF, (**B**) TGF-β1, (**C**) PDGF-BB, and (**D**) VEGF. There were no notable differences in the concentrations of BDNF, TGF-β1, and PDGF-BB between H-hPL and Fd-hPL. However, Fd-hPL exhibited a decreased concentration of VEGF compared to H-hPL. Values expressed as the mean ± SEM (*N* = 5 per group). * *p* < 0.05; unpaired *t*-test with the Holm–Šídák method.

After fibrinogen depletion, the concentrations of BDNF (153.87 ± 11.94 ng/mL in Fd-hPL and 159.27 ± 38.51 ng/mL in H-hPL), TGF-β1 (7668.06 ± 166.86 pg/mL in Fd-hPL and 7827.78 ± 130.81 pg/mL in H-hPL), and PDGF-BB (1140 ± 57.96 pg/mL in Fd-hPL and 1068.89 ± 57.34 pg/mL in H-hPL) were maintained. A significant decrease in the concentration was recorded for VEGF, dropping from 550.64 ± 174.11 pg/mL in H-hPL to 83.06 ± 26.68 pg/mL in Fd-HPL (Figure 3).

### 3.3. Morphology of UC-MSCs

**Figure 4:**
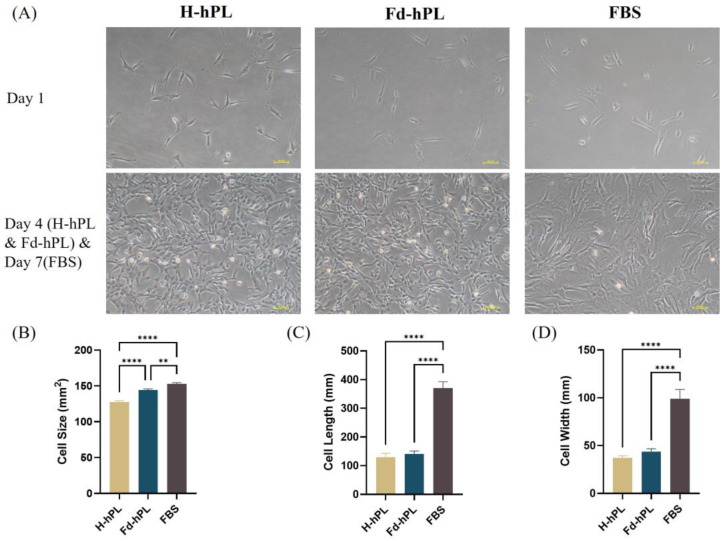
(**A**) Morphology of UC-MSCs cultured in different serum supplements (10% H-hPL, 10% Fd-hPL, and 10% FBS) under inverted microscopy. (**B**) Cell size of UC-MSCs cultured in different serum supplements. (**C**) The cell length of UC-MSCs cultured in various serum supplements was measured and compared. (**D**) Cell width of UC-MSCs cultured in different serum supplements. All groups exhibited comparable long spindle-shaped fibroblastic morphologies. Both H-hPL and Fd-hPL groups exhibited reduced cell size, cell length, and cell width in comparison to the FBS group. Values expressed as the mean ± SEM. ** *p* < 0.01 and **** *p* < 0.0001; one-way ANOVA with Tukey’s multiple comparison test.

UC-MSCs cultured with H-hPL and Fd-hPL took four days to reach 70%–80% confluency, whereas cells cultured with FBS needed seven days to reach the same confluency. The cells in all groups had similar spindle-shaped fibroblastic morphologies. However, UC-MSCs cultured in FBS displayed less consistent cell morphologies, with some cells exhibiting irregular flattened shapes and enlarged sizes (Figure 4A). In Figure 4B, the H-hPL group was smallest in cell size, followed by the Fd-hPL and FBS groups. Both hPL groups demonstrated similar cell lengths (Figure 4C) and widths (Figure 4D), while they were smaller in comparison to the FBS group.

### 3.4. UC-MSCs Grown in Fd-hPL Showed Typical MSC Surface Marker Expression Profiles

**Figure 5:**
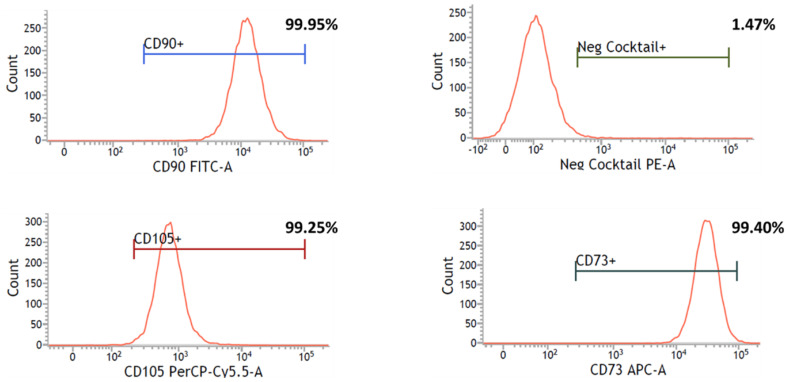
Flow cytometry analysis showed that UC-MSCs cultured with Fd-hPL expressed the MSC-specific markers CD73, CD90, and CD105, and were negative for the hematopoietic markers CD34, CD45, CD11b, CD14, CD19, and HLA-DR in negative cocktails.

Immunophenotyping was performed to determine the surface marker expression profile of UC-MSCs cultured with Fd-hPL. Figure 5 demonstrates that more than 99% of UC-MSCs were positive for CD73, CD90, and CD105, whilst less than 2% of the cells showed positive expression of negative markers CD34, CD45, CD11b, CD14, CD19, and HLA-DR.

### 3.5. UC-MSCs Grown in Fd-hPL Retained Adipogenic and Osteogenic Differentiation Potential

**Figure 6:**
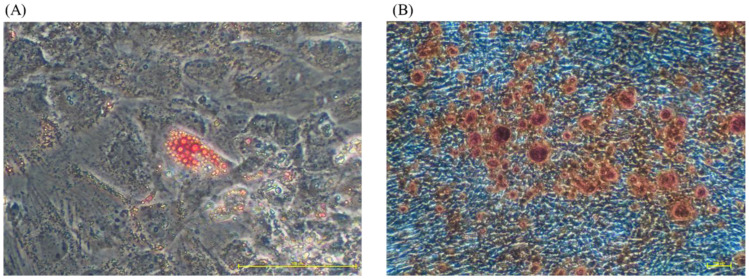
The multilineage differentiation potential of cultivated UC-MSCs. The UC-MSCs were cultured in adipogenic and osteogenic medium for two weeks. (**A**) Lipid droplets were detected in cytoplasm after a two-week induction using oil red O staining (under magnification 20×). (**B**) The presence of calcium deposition in the cells’ extracellular matrix was determined by Alizarin Red staining (under magnification 4×).

The multilineage differentiation potential of UC-MSCs cultured with Fd-hPL was determined using the adipogenic and osteogenic differentiation assays. After two weeks of culture in adipogenic differentiation medium, lipid droplets (red vesicles) were spotted in the cell cytoplasm using the oil red O stain (Figure 6A). Calcium deposition (red spots) was apparent upon two weeks of culture in osteogenic differentiation medium after Alizarin Red staining (Figure 6B).

### 3.6. Cell Viability, Total Cell Number, and PDT of UC-MSCs

**Figure 7:**
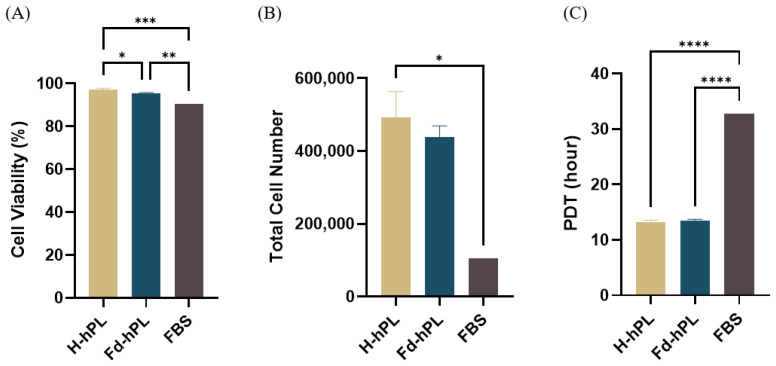
Viability, yield, and PDT of UC-MSCs cultured with different serum supplements. (**A**) All the groups demonstrated excellent viability exceeding 90%. (**B**) The total cell number was highest in the H-hPL group, followed by the Fd-hPL and FBS group. (**C**) The H-hPL and Fd-hPL groups had significantly shorter PDTs compared to the FBS group. Values expressed as the mean ± SEM (*N* = 6 for H-hPL and Fd-hPL and *N* = 1 (three technical replicates) for FBS). * *p* < 0.05, ** *p* < 0.01, *** *p* < 0.001, and **** *p* < 0.0001; one-way ANOVA with Tukey’s multiple comparison test.

UC-MSCs were trypsinized once they reached 70%–80% confluency to determine the viability, cell yield, and PDT. The UC-MSCs cultured with H-hPL and Fd-hPL were trypsinized at day 4 and the cells cultured with FBS were trypsinized at day 7. The cell viability exceeded 90% for all groups with the H-hPL (97.28 ± 0.56%) having the highest cell viability, followed by Fd-hPL (95.44 ± 0.36%) and FBS (90.48%) (Figure 7A). Starting with a seeding density of 3000 cells/cm^2^ in a six-well plate, a total of 492,500, 425,833, and 105,000 cells were obtained in the H-hPL, Fd-hPL, and FBS groups, respectively. The PDT of the UC-MSCs grown in H-hPL (13.21 ± 0.33 h) and Fd-hPL (13.49 ± 0.22 h) was significantly shorter compared to the UC-MSCs grown in FBS (32.75 h) (Figure 7).

## 4. Discussion

The use of FBS for ex vivo expansion of MSCs to be applied clinically for disease modification will need reconsideration as the cultured cells will contain xenogeneic proteins that may activate the recipient’s immune system to reject the transplanted cells [23]. Additionally, they contain endotoxins, which increase the risk of zoonotic disease transmission. Due to these limitations, human-derived alternatives such as platelet-rich plasma, human serum, and hPL are being used as safer alternatives for MSC culture in the current good manufacturing practice (cGMP) facility [1,24]. Currently, hPL seems to be the most ideal substitute for FBS as it is rich in platelet-specific growth factor, can efficiently increase MSC proliferation without compromising cell functionality, and it is very safe with no serious side effects reported in clinical studies. However, crude hPL is rich in fibrinogen, thrombocyte-derived factors, and clotting factors that significantly escalate the possibility of fibrin clot formation [25,26,27]. The presence of this gel layer might hamper cell growth and interfere in the cell harvesting process. Typically, animal-derived heparin is added to the culture medium to prevent clot formation. Even so, it cannot eliminate the risk of clot formation, and increasing the heparin concentration is not a viable option as it is known to be cytotoxic at high concentrations. High concentrations of heparin will lead to cell growth inhibition and apoptosis [28,29]. More alarmingly, porcine heparin has been linked with serious side effects such as thrombocytopenia, hypersensitivity, and hyperkalemia in up to 5% of patients [30,31,32]. Hence, this study aimed to identify a reliable and effective approach for preparing hPL that is clot-free even without the addition of heparin for efficient expansion of MSCs. UC-MSCs were selected as the model cell for this study due to their inherent advantages in the field of regenerative medicine and cell therapy. These advantages include their widespread availability, a non-invasive isolation process that is ethically acceptable, a higher capacity for expansion compared to adult MSCs, and robust immunomodulatory capabilities [33,34]. These characteristics made UC-MSCs an ideal choice for investigating the effects and potential applications of Fd-hPL in this study.

The freeze–thaw cycle method was chosen for the preparation of hPL due to its widespread use and reported advantages, including simplicity and cost-effectiveness [10,25,27]. Previous studies have consistently demonstrated the effectiveness of hPL prepared using this technique. These studies have shown that cells cultured with hPL exhibit a significantly higher cell yield and shorter PDT compared to cells expanded with FBS [17,18,19,20]. The process of removing fibrinogen was carried out using a calcium salt solution to stimulate platelet activation and coagulation [35]. hPL became clear and precipitate-free upon calcium salt treatment due to fibrinogen removal. Even though the concentration of fibrinogen did not reduce significantly, it was low enough to avoid the need for heparin to prevent clot formation after multiple freeze-thaw cycles. Apart from preventing clot formation, low fibrinogen levels might also improve the MSC therapeutic potential as prior studies have revealed that the presence of fibrinogen increases the secretion of pro-inflammatory cytokines such as MCP-1, IL-8, and IL-6 by MSCs, human dendritic cells, endothelial cells, and pancreatic stellate cells [25,36,37,38,39]. Copland et al. showed that fibrinogen also negatively impacts the immunosuppressive capabilities of MSCs [25]. Calcium salt treatment did increase the calcium levels in Fd-hPL. However, this does not jeopardize the safety or efficacy of cells cultured with Fd-hPL [11,25].

HPL is rich in growth factors that are critical for stimulating cell proliferation and maintaining cell metabolism [40]. Thus, it is important to determine the concentration of the important growth factors upon fibrinogen depletion as they may be removed together with the fibrin matrix. In this study, we measured the concentration of four growth factors—PDGF, VEGF, BDNF, and TGF- β1. Only the concentration of VEGF dropped significantly after calcium treatment, whilst the concentrations of other growth factors were maintained. The VEGF level was reduced as it binds fibrinogen and fibrin with high affinity in a unique and saturable manner [41]. The unchanged levels of TGF-β1 and BDNF is quite surprising as Martino et al. mentioned that BDNF and TGF-β1 have high binding affinity to fibrinogen, which leads to retention in the fibrin matrix [42]. PDGF also showed binding affinity to fibrin. However, the unchanged levels of PDGF found in this study could be due to the rapid release of PDGF from the matrix. In the same study, the authors also found that fibrin binds with growth factors from the VEGF/PDGF and FGF families through its heparin-binding domain [42].

The findings from the cell culture experiments showed that a high calcium level and a lower concentration of VEGF in Fd-hPL does not affect the growth and characteristics of MSCs as no significant changes in cell yield, PDT, multilineage differentiation potential, or surface marker expression were recorded. These results are consistent with the findings of a previous study that mentioned that a high calcium ion concentration could only maintain but failed to enhance cell growth [43]. In addition, the elevated calcium level in Fd-hPL (45.10 ± 3.89 nmole/mL) was still lower than the calcium level in FBS, which varied from 3.5 to 4 mM [44]. A mild drop in the total cell number in the Fd-hPL group might be due to the slight reduction in the concentration of PDGF, which is essential for improving the development and survival of a variety of cell types, as well as encouraging the proliferation of MSCs [45,46]. Moreover, the significantly lowered VEGF levels might also be a contributing factor, because VEGF can activate PDGF receptors, which control the proliferation and migration of MSCs [47]. Importantly, the MSCs expanded with Fd-hPL retained the MSC characteristics as indicated by their fibroblastic morphology, adipogenic and osteogenic differentiation potential, and positive expression of CD73, CD90, and CD105, along with the negative expression of CD34, CD45, CD11b, CD14, CD19, and HLA-DR. This outcome is in line with the findings of Copland et al. [25].

In a nutshell, our study suggested that Fd-hPL has the potential to be an alternative for H-hPL to cultivate UC-MSCs in vitro. However, further studies are needed to fully comprehend the molecular processes underlying the interaction between the growth factors, fibrinogen, and heparin and the biological activity of MSCs. Additionally, the use of hPL in cell cultures is associated with batch-to-batch variation. Thus, efforts should be made to minimize these variations. Moreover, it is possible to assess the impact of hPL on other cell types in the future to identify its potential uses and limitations as the cells may respond differently to Fd-HPL. Evaluating the effects of Fd-HPL on these cell types can help determine its potential applications in tissue engineering, wound healing, and other therapeutic interventions.

## 5. Conclusions

We successfully prepared Fd-hPL that does not clot in cultures by using calcium salt to reduce the fibrinogen concentration. Fd-hPL demonstrated comparable efficacy as H-hPL in supporting MSC expansion in vitro without compromising the cell characteristics. However, the impact of changes in the fibrinogen, calcium, and growth factor concentrations on the cell functionality and therapeutic potential has yet to be fully elucidated. Therefore, more studies need to be performed to determine the possibility of using Fd-hPL to expand the MSCs in the cGMP facility for clinical applications.

## Data Availability

The data presented in this study are available on request from the corresponding author.

## References

[1] Guiotto M., Raffoul W., Hart A.M., Riehle M.O., Di Summa P.G. (2020). Human Platelet Lysate to Substitute Fetal Bovine Serum in HMSC Expansion for Translational Applications: A Systematic Review. J. Transl. Med..

[2] Hassan M.N.F.B., Yazid M.D., Yunus M.H.M., Chowdhury S.R., Lokanathan Y., Idrus R.B.H., Ng A.M.H., Law J.X. (2020). Large-Scale Expansion of Human Mesenchymal Stem Cells. Stem Cells Int..

[3] Mackensen A., Dräger R., Schlesier M., Mertelsmann R., Lindemann A. (2000). Presence of IgE Antibodies to Bovine Serum Albumin in a Patient Developing Anaphylaxis after Vaccination with Human Peptide-Pulsed Dendritic Cells. Cancer Immunol. Immunother..

[4] Dessels C., Potgieter M., Pepper M.S. (2016). Making the Switch: Alternatives to Fetal Bovine Serum for Adipose-Derived Stromal Cell Expansion. Front. Cell Dev. Biol..

[5] Bieback K., Fernandez-Muñoz B., Pati S., Schäfer R. (2019). Gaps in the Knowledge of Human Platelet Lysate as a Cell Culture Supplement for Cell Therapy: A Joint Publication from the AABB and the International Society for Cell & Gene Therapy. Transfusion.

[6] National Blood Centre (2016). Transfussion Practice Guidelines for Clinical and Laboratory Personnel.

[7] Aubron C., Flint A., Ozier Y., McQuilten Z. (2016). Transfusion of Stored Platelets: Balancing Risks and Product Availability. Int. J. Clin. Transfus. Med..

[8] Dessels C., Durandt C., Pepper M.S. (2019). Comparison of Human Platelet Lysate Alternatives Using Expired and Freshly Isolated Platelet Concentrates for Adipose-Derived Stromal Cell Expansion. Platelets.

[9] Glovinski P.V., Herly M., Mathiasen A.B., Svalgaard J.D., Borup R., Talman M.L.M., Elberg J.J., Kølle S.F.T., Drzewiecki K.T., Fischer-Nielsen A. (2017). Overcoming the Bottleneck of Platelet Lysate Supply in Large-Scale Clinical Expansion of Adipose-Derived Stem Cells: A Comparison of Fresh versus Three Types of Platelet Lysates from Outdated Buffy Coat–Derived Platelet Concentrates. Cytotherapy.

[10] Bianchetti A., Chinello C., Guindani M., Braga S., Neva A., Verardi R., Piovani G., Pagani L., Lisignoli G., Magni F. (2021). A Blood Bank Standardized Production of Human Platelet Lysate for Mesenchymal Stromal Cell Expansion: Proteomic Characterization and Biological Effects. Front. Cell Dev. Biol..

[11] Delabie W., De Bleser D., Vandewalle V., Vandekerckhove P., Compernolle V., Feys H.B. (2022). Single Step Method for High Yield Human Platelet Lysate Production. Transfusion.

[12] Astori G., Amati E., Bambi F., Bernardi M., Chieregato K., Schäfer R., Sella S., Rodeghiero F. (2016). Platelet Lysate as a Substitute for Animal Serum for the Ex-Vivo Expansion of Mesenchymal Stem/Stromal Cells: Present and Future. Stem Cell Res. Ther..

[13] da Fonseca L., Santos G.S., Huber S.C., Setti T.M., Setti T., Lana J.F. (2021). Human Platelet Lysate—A Potent (and Overlooked) Orthobiologic. J. Clin. Orthop. Trauma.

[14] Jinhee H., Stephanie A.S.-W., Eric B.R., Walter C.W., Wu K., Yin C.E.G. (2017). Effect of Heparin on the Biological Properties and Molecular Signature of Human Mesenchymal Stem Cells. J. Int. Soc. Burn Inj..

[15] Furue M.K., Na J., Jackson J.P., Okamoto T., Jones M., Baker D., Hata R.I., Moore H.D., Sato J.D., Andrews P.W. (2008). Heparin Promotes the Growth of Human Embryonic Stem Cells in a Defined Serum-Free Medium. Proc. Natl. Acad. Sci. USA.

[16] Mimura S., Kimura N., Hirata M., Tateyama D., Hayashida M., Umezawa A., Kohara A., Nikawa H., Okamoto T., Furue M.K. (2011). Growth Factor-Defined Culture Medium for Human Mesenchymal Stem Cells. Int. J. Dev. Biol..

[17] Hassan M.N.F., Yap Z.Y., Tang Y.L., Ng M.H., Law J.X. (2021). Expired Platelet Concentrate as a Source of Human Platelet Lysate for Xenogeneic-Free Culture of Human Dermal Fibroblasts. Sains Malaysiana.

[18] Liau L.L., Hassan M.N.F., Tang Y.L., Ng M.H., Law J.X. (2021). Feasibility of Human Platelet Lysate as an Alternative to Foetal Bovine Serum for in Vitro Expansion of Chondrocytes. Int. J. Mol. Sci..

[19] Budi Harto P.H., Muhammad Hanif M., Othman A.H., Ngadenin N.H., Mohd Azahar N.S., Hassan M.N.F., Mohd Yahaya N.H., Rani R.A., Leong C.F., Ng M.H. (2019). Human Platelet Lysate Promotes Proliferation but Fails to Maintain Chondrogenic Markers of Chondrocytes. Sains Malaysiana.

[20] Chan A.M.L., Ng A.M.H., Mohd Yunus M.H., Hj Idrus R., Law J.X., Yazid M.D., Chin K.Y., Shamsuddin S.A., Mohd Yusof M.R., Razali R.A. (2022). Safety Study of Allogeneic Mesenchymal Stem Cell Therapy in Animal Model. Regen. Ther..

[21] Lipinski B., Pretorius E. (2012). Novel Pathway of Iron-Induced Blood Coagulation: Implications for Diabetes Mellitus and Its Complications. Pol. Arch. Med. Wewn..

[22] Dominici M., Le Blanc K., Mueller I., Slaper-Cortenbach I., Marini F.C., Krause D.S., Deans R.J., Keating A., Prockop D.J., Horwitz E.M. (2006). Minimal Criteria for Defining Multipotent Mesenchymal Stromal Cells. The International Society for Cellular Therapy Position Statement. Cytotherapy.

[23] Shi Y., Hu G., Su J., Li W., Chen Q., Shou P., Xu C., Chen X., Huang Y., Zhu Z. (2010). Mesenchymal Stem Cells: A New Strategy for Immunosuppression and Tissue Repair. Cell Res..

[24] Even M.S., Sandusky C.B., Barnard N.D. (2006). Serum-Free Hybridoma Culture: Ethical, Scientific and Safety Considerations. Trends Biotechnol..

[25] Copland I.B., Garcia M.A., Waller E.K., Roback J.D., Galipeau J. (2013). The Effect of Platelet Lysate Fibrinogen on the Functionality of MSCs in Immunotherapy. Biomaterials.

[26] Laner-Plamberger S., Lener T., Schmid D., Streif D.A., Salzer T., Öller M., Hauser-Kronberger C., Fischer T., Jacobs V.R., Schallmoser K. (2015). Mechanical Fibrinogen-Depletion Supports Heparin-Free Mesenchymal Stem Cell Propagation in Human Platelet Lysate. J. Transl. Med..

[27] Hemeda H., Giebel B., Wagner W. (2014). Evaluation of Human Platelet Lysate versus Fetal Bovine Serum for Culture of Mesenchymal Stromal Cells. Cytotherapy.

[28] Mohamed H.E., Asker M.E., Kotb N.S., El Habab A.M. (2020). Human Platelet Lysate Efficiency, Stability, and Optimal Heparin Concentration Required in Culture of Mammalian Cells. Blood Res..

[29] Gurbuz H.A., Durukan A.B., Sevim H., Ergin E., Gurpinar A., Yorgancioglu C. (2013). Heparin Toxicity in Cell Culture: A Critical Link in Translation of Basic Science to Clinical Practice. Blood Coagul. Fibrinolysis.

[30] Linkins L.A. (2015). Heparin Induced Thrombocytopenia. BMJ.

[31] Gonzalez-Delgado P., Fernandez J. (2016). Hypersensitivity Reactions to Heparins. Curr. Opin. Allergy Clin. Immunol..

[32] Talib U., Lee P. (2019). Heparin-Induced Hyperkalemia: The Un-Noticed Shadow. Chest.

[33] Selich A., Zimmermann K., Tenspolde M., Dittrich-Breiholz O., Von Kaisenberg C., Schambach A., Rothe M. (2019). Umbilical Cord as a Long-Term Source of Activatable Mesenchymal Stromal Cells for Immunomodulation. Stem Cell Res. Ther..

[34] El Omar R., Beroud J., Stoltz J.F., Menu P., Velot E., Decot V. (2014). Umbilical Cord Mesenchymal Stem Cells: The New Gold Standard for Mesenchymal Stem Cell-Based Therapies?. Tissue Eng.-Part B Rev..

[35] Burnouf T., Strunk D., Koh M.B.C., Schallmoser K. (2016). Human Platelet Lysate: Replacing Fetal Bovine Serum as a Gold Standard for Human Cell Propagation?. Biomaterials.

[36] Salam N., Toumpaniari S., Gentile P., Ferreira A.M., Dalgarno K., Partridge S. (2018). Assessment of Migration of Human MSCs through Fibrin Hydrogels as a Tool for Formulation Optimisation. Materials.

[37] Guo M., Sahni S.K., Sahni A., Francis C.W. (2004). Fibrinogen Regulates the Expression of Inflammatory Chemokines through NF-ΚB Activation of Endothelial Cells. Thromb. Haemost..

[38] Thacker R.I., Retzinger G.S. (2008). Adsorbed Fibrinogen Regulates the Behavior of Human Dendritic Cells in a CD18-Dependent Manner. Exp. Mol. Pathol..

[39] Masamune A., Kikuta K., Watanabe T., Satoh K., Hirota M., Hamada S., Shimosegawa T. (2009). Fibrinogen Induces Cytokine and Collagen Production in Pancreatic Stellate Cells. Gut.

[40] Oeller M., Laner-plamberger S., Krisch L., Rohde E., Strunk D., Schallmoser K. (2021). Human Platelet Lysate for Good Manufacturing Practice-compliant Cell Production. Int. J. Mol. Sci..

[41] Sahni A., Francis C.W. (2000). Vascular Endothelial Growth Factor Binds to Fibrinogen and Fibrin and Stimulates Endothelial Cell Proliferation. Blood.

[42] Martino M.M., Briquez P.S., Ranga A., Lutolf M.P., Hubbell J.A. (2013). Heparin-Binding Domain of Fibrin(Ogen) Binds Growth Factors and Promotes Tissue Repair When Incorporated within a Synthetic Matrix. Proc. Natl. Acad. Sci. USA.

[43] Liu Y.K., Lu Q.Z., Pei R., Ji H.J., Zhou G.S., Zhao X.L., Tang R.K., Zhang M. (2009). The Effect of Extracellular Calcium and Inorganic Phosphate on the Growth and Osteogenic Differentiation of Mesenchymal Stem Cells in Vitro: Implication for Bone Tissue Engineering. Biomed. Mater..

[44] Blankenship J.R., Heitman J. (2005). Calcineurin Is Required for *Candida albicans* to Survive Calcium Stress in Serum. Infect. Immun..

[45] Zhang J., Feng F., Wang Q., Zhu X., Fu H., Xu L., Liu K., Huang X., Zhang X. (2016). Platelet-Derived Growth Factor-BB Protects Mesenchymal Stem Cells (MSCs) Derived From Immune Thrombocytopenia Patients Against Apoptosis and Senescence and Maintains MSC-Mediated Immunosuppression. Tissue-Specific Progenit. Stem Cellls.

[46] Qiu P., Song W., Niu Z., Bai Y., Li W., Pan S., Peng S., Hua J. (2013). Platelet-Derived Growth Factor Promotes the Proliferation of Human Umbilical Cord-Derived Mesenchymal Stem Cells. Cell Biochem. Funct..

[47] Ball S.G., Shuttleworth C.A., Kielty C.M. (2007). Mesenchymal Stem Cells and Neovascularization: Role of Platelet-Derived Growth Factor Receptors. J. Cell. Mol. Med..

